# Effectiveness of Casein Phosphopeptide-Amorphous Calcium Phosphate (CPP-ACP) Compared to Fluoride Products in an In-Vitro Demineralization Model

**DOI:** 10.3390/ma14205974

**Published:** 2021-10-11

**Authors:** Markus Reise, Stefan Kranz, Markus Heyder, Klaus D. Jandt, Bernd W. Sigusch

**Affiliations:** 1 Department of Conservative Dentistry and Periodontology, Jena University Hospital, An der Alten Post 4, 07743 Jena, Germany; Stefan.Kranz@med.uni-jena.de (S.K.); Markus.Heyder@med.uni-jena.de (M.H.); Bernd.W.Sigusch@med.uni-jena.de (B.W.S.); 2 Chair of Materials Science, Otto Schott Institute of Materials Research (OSIM), Faculty of Physics and Astronomy, Friedrich Schiller University Jena, Löbdergraben 32, 07743 Jena, Germany; Klaus.D.Jandt@uni-jena.de

**Keywords:** casein phosphopeptide amorphous calcium phosphate, amorphous calcium fluoride phosphate, amine fluoride, sodium fluoride, demineralization, remineralization, enamel, dentin, dental caries, erosion, white spot

## Abstract

The goal of this study was to evaluate the effectiveness of the toothpaste Tooth Mousse compared to conventional fluoride-based versions in the prevention of enamel and dentin demineralization. Human enamel and dentin samples (*n* = 120 each) were exposed to artificial demineralization at pH 4.92. During the demineralization process, the samples in the test groups were periodically treated with Tooth Mousse (TM) containing casein-phosphopeptide -amorphous-calcium-phosphate (CPP-ACP) and Tooth Mousse Plus (TMP) containing amorphous-calcium-fluoride-phosphate (CPP-ACPF) to evaluate their protective properties. Fluoride toothpastes containing 1400 ppm amine fluoride (AmF) and 1450 ppm sodium fluoride (NaF) were applied in the positive control groups. Treatment with distilled water (group C-W) or demineralization without treatment (group C-D) served as negative controls. After the demineralization and treatment process, all samples were cut longitudinally and lesion depths were determined at six locations using polarized light microscopy. In TM/TMP groups (enamel: 80/86 µm, dentin: 153/156 µm) lesion depths were significantly smaller compared to the negative control groups C-W/C-D (enamel: 99/111 µm, dentin: 163/166 µm). However, TM and TMP compared to the positive controls AmF/NaF (enamel: 58/63 µm, dentin: 87/109 µm) showed higher lesion depths. The application of TM/TMP (89%/78%) during demineralization led to a reduced number of severe lesions compared to the negative controls C-W/C-D (100%/95%). In this study we demonstrate that Tooth Mousse is less effective regarding prevention of enamel and dentin demineralization compared to fluoride containing toothpastes.

## 1. Introduction

Dental enamel and dentin are the hardest tissues in the human body. Nevertheless, they are susceptible to the impact of different acids. Depending on the origin of the acids, a differentiation is made between dental decay caused by organic acids produced by bacteria, i.e., dental caries, and destruction of dental tissues by acids that are not related to the metabolism of bacteria, i.e., dental erosion. However, both phenomena represent major issues in dentistry [1,2,3].

The focus in the prevention of caries and erosion has been based on the application of fluoride [3,4,5,6,7]. Alternative approaches are also being frequently discussed [2,8]. One class of agents with potential caries-reducing properties includes milk-protein-based complexes. The first scientific reports about the anti-carious effects of the milk protein casein were published as early as 1946 by Schweigert et al. [9]. Further development led to the synthesis of specific peptides [10,11,12]. The most investigated casein-based peptide complex is casein phosphopeptide-amorphous calcium phosphate (CPP-ACP) [8,13,14,15,16]. Due to the enzymatic breakdown of α_s1_-casein by trypsin, CPP has several advantages. Compared to pure α_s1_-casein, CPP also acts less allergenic [17,18]. According to Reynolds et al. the anti-cariogenic activity of CPP-ACP is based on the ability of CPP to stabilize calcium and phosphate ions in a metastable solution [11]. Analogous to this system, stable complexes with fluoride ions like CPP amorphous calcium fluoride phosphate (phosphopeptide-amorphous calcium phosphate CPP-ACFP) were also generated [19,20]. Additionally, CPP-ACP and CPP-ACFP might function as buffer systems which reduce the destructive impact of acids [21].

In recent years, several investigations have focused on the effectiveness of CPP-ACP regarding the prophylaxis and treatment of dental caries and erosion as well as the treatment of white spot lesions [22,23].

In some in vitro and in vivo studies, a preventive effect on early dental caries lesions was observed to be caused by the remineralization potential of CPP-ACP [12,24,25,26]. However, other studies indicate only minor or no benefit of CPP-ACP products for the treatment of carious damaged enamel [15,21,27]. In a recently published systemic review by Puleio et al. [22], it was stated that the treatment of white spot lesions with CPP-ACP is limited to early stages since its infiltrating capacity is limited.

If CPP-ACP shows comparable demineralization protective qualities as fluoride toothpastes, it might be used in many fields of preventive dentistry and daily oral hygiene.

Therefore, the aim of the present in vitro study was to evaluate the protective influence of CPP-ACP/CPP-ACPF containing products on the demineralization process of human enamel and dentin samples in comparison to commercially available fluoride-containing toothpastes.

## 2. Materials and Methods

**Figure 1:**
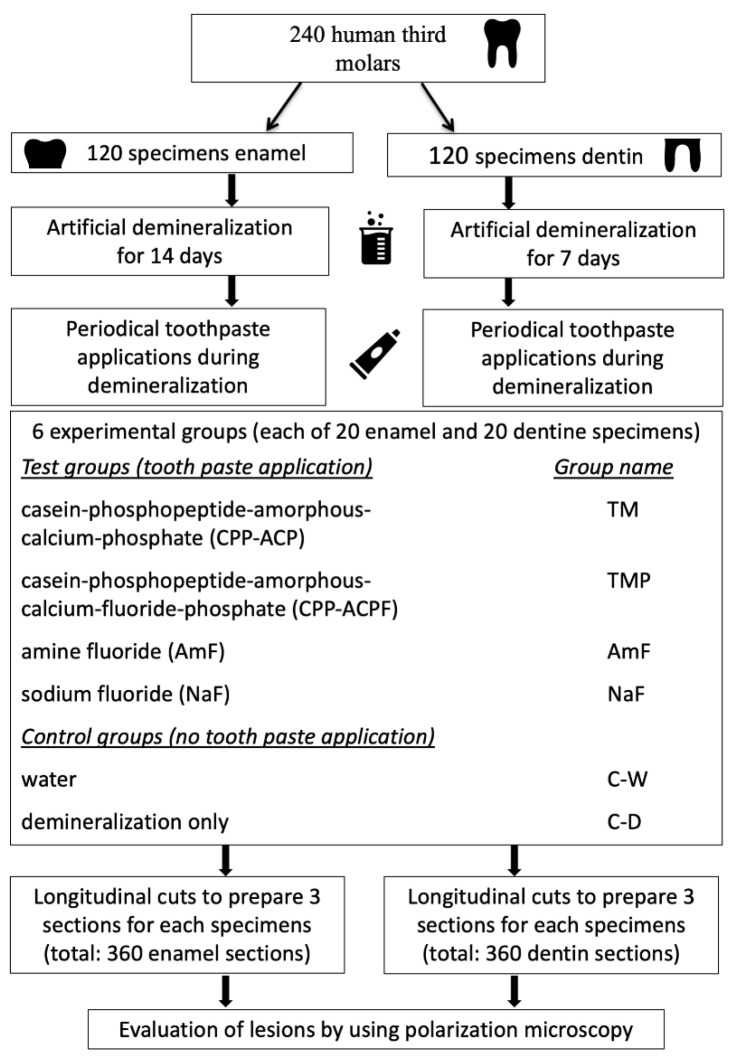
Schematic overview illustrating the experimental procedure of this study.

To obtain enamel and dentin samples, 240 caries-free human teeth (third molars/approved by the Ethics Committee Jena # B2428-10/08; Ethics Committee of Friedrich-Schiller-University Jena at the Medical Faculty, Bachstrasse 18, 07740 Jena, Germany) were carefully cleaned from soft tissues and stored in 0.1% thymol solution until preparation of the specimens. Stereomicroscopy (Stemi 2000 C, Carl Zeiss Microlmaging GmbH, Jena, Germany) was performed to examine the teeth for any defects and to determine suitable surfaces for the study. The selected surfaces were polished using polishing paste (Clean Polish/Super Polish, Kerr, Bioggio, Switzerland). Dispensable crown and root fragments were removed by using a rotating dental burr with under cooling (50 mL/min). The specimens were stored in distilled water until they were used for further investigation. The experimental procedure of this in vitro study is shown in Figure 1.

### 2.1. Experimental Demineralization

In this in vitro study enamel and dentin samples (*n* = 120 each) from caries-free extracted third molars were covered with modelling wax (Schuler-Dental GmbH und Co. KG, Ulm, Germany). To achieve a standardized evaluation of the lesions, a wax-free test area (≥2.5 mm length/1.2–1.5 mm width) was created in the middle of the outer enamel surfaces). For dentine lesions, the wax-free area was created in the coronal third of the root.

Enamel specimens were exposed to a demineralization solution (DS) for 14 days, and dentine specimens for 7 days. In both experimental arrangements (enamel and dentin) the DS was replaced daily. After 7 days, the DS was completely renewed.

During the experimental demineralization all specimens were stored at 37 °C (Incubator: BED 53, WTB Binder Labortechnik GmbH, Tuttlingen, Germany) in a solution composed of 0.1 molar sodium acetate buffer in 6% hydroxyethylcellulose (HEC) at pH 4.92 ± 0.02 (pH-meter: pH 211, HANNA-Instruments GmbH, Kehl am Rhein, Germany), supplemented with 0.9 mmol/L KH_2_PO_4_ and 150 mmol/L CaCl_2_ [28].

### 2.2. Experimental Groups

This study aims to determine the protective influence of CPP-ACP/ACPF toothpaste used during the demineralization process of enamel and dentin in vitro. Two products containing CPP-ACP were compared to two common commercially available toothpastes based on amine fluoride (AmF) and sodium fluoride (NaF). Six experimental groups (*n* = 20/group) were created:

Test groups:

Group TM: CPP-ACP (GC Tooth Mousse, GC EUROPE N.V., Leuven, Belgium);

Group TMP: CPP-ACP with 900 ppm NaF (GC MI Paste Plus, GC EUROPE N.V., Leuven, Belgium).

Positive control groups:

Group AmF: 1400 ppm amine fluoride (Elmex Caries Protection, CP GABA, NY, USA); Group NaF: 1450 ppm NaF (Sensodyne Pronamel, GSK House, Brentford, UK).

Negative control groups:

Group C-W: distilled water; baseline;

Group C-D: demineralization only (no treatment with toothpastes at all).

### 2.3. Application of Test Agents

**Figure 2:**
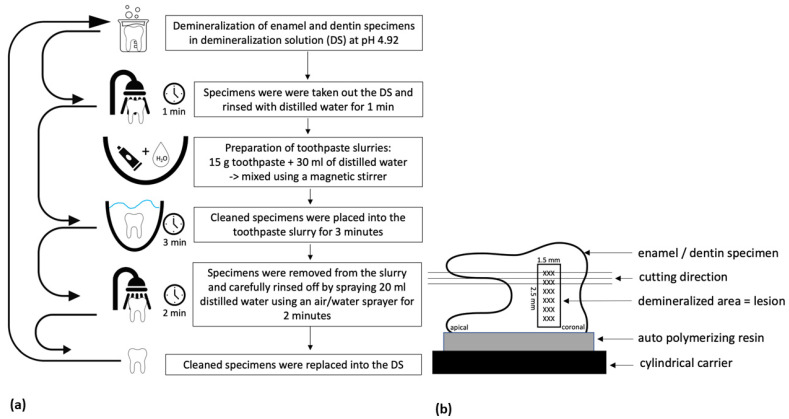
(**a**). Flowchart of an application cycle illustrating the sequence of the experimental demineralization and application of the test agents. (**b**) The principle of preparing three longitudinal sections for each lesion for further microscopically evaluation.

All experiments were performed under standard laboratory conditions at a room temperature of 20 °C. Toothpaste slurries were prepared daily containing 15 g of the test agent and 30 mL of distilled water (Figure 2a).

Both ingredients were mixed to a homogeneous slurry using a magnetic stirrer. Before application of the test slurries, specimens were taken out of the demineralization solution (DS) and rinsed with distilled water for 1 min. Cleaned specimens were placed into the test slurry for 3 min while the slurry was stirred with the magnetic stirrer. After this procedure, the specimens were removed from the slurry and were carefully rinsed off with 20 mL distilled water via an air/water sprayer for 2 min. The specimens were then placed back into the DS.

In total, 19 applications of test slurries were performed for enamel during a demineralization period of 14 days and 9 days for the dentin specimens during a demineralization period of 7 days.

For control group C-W 40 mL of distilled water was used instead of toothpaste slurry. Specimens from control group C-D were stored in the DS over the entire experimental period.

At the end of the study period, the specimens were taken out of the DS, rinsed with 20 mL distilled water for 1 min and the protection wax was removed. The DS was changed after each application cycle.

### 2.4. Section Preparation

Preparation of plane-parallel thin-sections was necessary to evaluate initial demineralized lesions in polarized light (Figure 2b). The specimens were fixed to a cylindrical carrier using auto-polymerizing resin (Kallocryl CPGM rot, SPEIKO^®^-Dr. Speier GmbH, Münster, Germany) and adjusting the lesion perpendicular to the sawing blade. For each specimen three longitudinal sections (80–110 µm) through the central part of the lesion were prepared with constant water-cooling using a low-speed saw microtome Leitz 1600 (Leica Mikrosysteme Vertrieb GmbH, Wetzlar, Germany). The sections were placed onto microscope slides and protected with cover slips. In total, 360 sections each of enamel and dentin were generated.

Each test was performed on an individual enamel or dentin specimen to obtain adequately sized samples for further evaluation. This also helped avoid possible interactions of active agents in the control samples caused by ion exchange.

### 2.5. Polarized Light Microscopy

Evaluation and measurements of the sections were performed by a blinded and trained examiner using a JENAPOL polarized light microscope (Carl Zeiss Microlmaging GmbH, Jena, Germany). Photographic documentation and analysis were carried out by means of the high-resolution digital microscopy camera AxiaCam MRc5 (Carl Zeiss MicroImaging GmbH, Jena, Germany) and the AxioVision Rel. 4.6 software (enlargement M 140:1). Ethanol (100%) was used as the inhibition medium for enamel and distilled water for dentin. For enamel, lesion depth and thickness of the surface layer served as quantitative criteria. Measurements were performed on six locations of each individual lesion from which the average values were calculated. Additional qualitative assessment was performed to characterize the lesions ranging from defects showing only signs of demineralization in terms of increased delineation of enamel prisms and areas with positive birefringence over moderate lesions up to pronounced lesions. For dentin, the average lesion depth (from 3 to 6 measuring points) served as quantitative criteria. Furthermore, lamellar demineralization zones inside the lesions were documented. Besides the determination of the lesion depth, enamel lesions were also assigned to three categories assessed by the severity of demineralization: category 1: small signs of demineralization; category 2: moderate enamel lesion; and category 3: pronounced enamel lesion.

### 2.6. Statistical Analysis

Statistical analysis was performed using SPSS version 17 (SPSS Inc., Chicago, Illinois, USA) for Microsoft Windows 7 Professional for the six tested enamel and six dentin groups separately. For all statistical tests the significance level was set to α = 0.05 (*p*-value ≤0.05). To compare the mean values of all test groups, an analysis of variance (ANOVA) without repeated measurements was conducted. Further, a Shapiro-Wilk test was performed to determine normal distribution. The degree of variance homogeneity was evaluated using the Levene-test. To detect group-specific differences for the dentin test groups, a univariate ANOVA with a post-hoc multiple comparison (parametric test) was used, since ANOVAs are insensitive to small deviations from the normal distribution, which were detected only in group C-W.

In case of variance homogeneity, the Tukey test (for dentin groups) was conducted. For enamel groups (no variance homogeneity) the Games–Howell test was applied.

## 3. Results

### 3.1. Enamel Lesions

Enamel lesion depths observed after artificial demineralization and treatment with Tooth Mousse and Tooth Mousse Plus (TM: 86 ± 14 µm; TMP: 80 ± 14 µm) were in most cases significantly smaller than in the control groups (C-W: 99 ± 18 µm; CD: 111 ± 23 µm).

**Figure 3:**
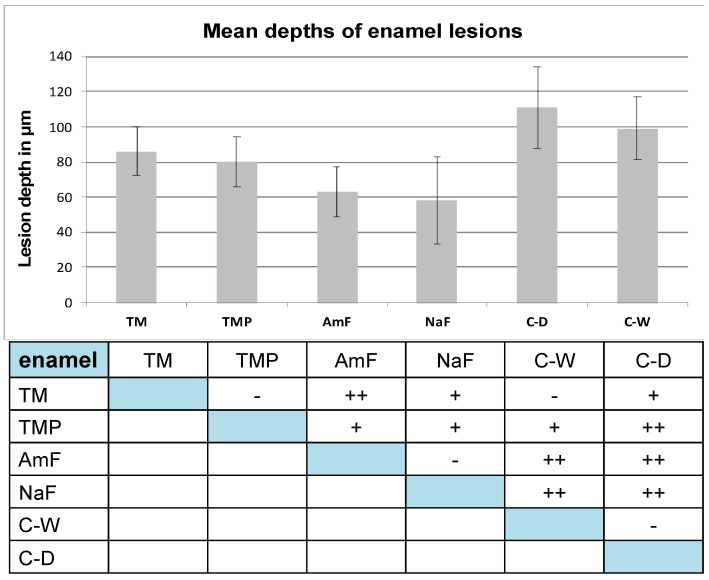
Mean lesion depths and standard deviations of enamel lesions. Below the statistical evaluation of lesion depths regarding significance between the different test groups for enamel groups: − not significant (*p* ≥ 0.05); + significant (*p* ≤ 0.01); ++ highly significant (*p* ≤ 0.001). Data were analyzed by ANOVA i.a.

However, compared to the test groups TM and TMP smaller lesion depths were measured in both fluoride-toothpaste positive control groups: group AmF and group NaF (63 ± 14 µm; 58 ± 14 µm) (see Figure 3).

### 3.2. Dentin Lesions

The dentin lesion depths which were detected in the test groups TM and TMP (156 ± 9 µm; 153 ± 8 µm) were also smaller than in the negative control groups (C-W: 166 ± 10 µm; C-D: 163 ± 14 µm).

**Figure 4:**
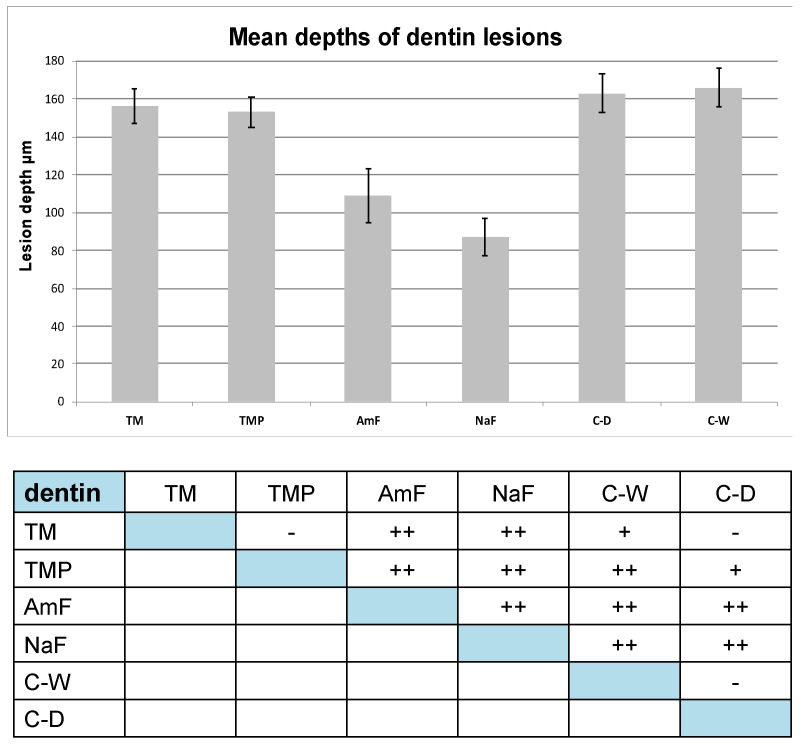
Mean lesion depths and standard deviations of dentin lesions. Below the statistical evaluation of lesion depths regarding significance between the different test groups for dentin groups: − not significant (*p* ≥ 0.05); + significant (*p* ≤ 0.01); ++ highly significant (*p* ≤ 0.001). Data were analyzed by ANOVA i.a.

Similar as observed in the enamel samples, smaller dentin lesion depths were found in the positive control groups AmF and NaF (109 ± 14 µm; 87 ± 10 µm) compared to the test groups (see Figure 4).

### 3.3. Morphological Evaluation of the Lesions

**Figure 5:**
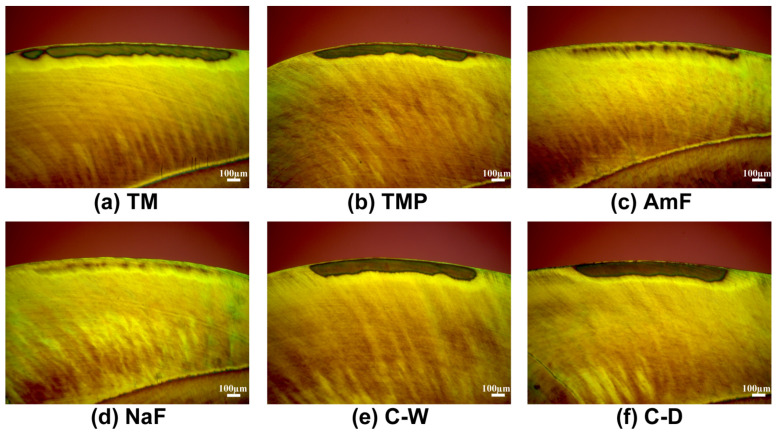
Polarized light images of enamel lesions showing different lesion depths caused by the protective influence of treatment with different toothpaste slurries (**a**–**d**) during artificial demineralization compared to controls (**e**,**f**).

**Figure 6:**
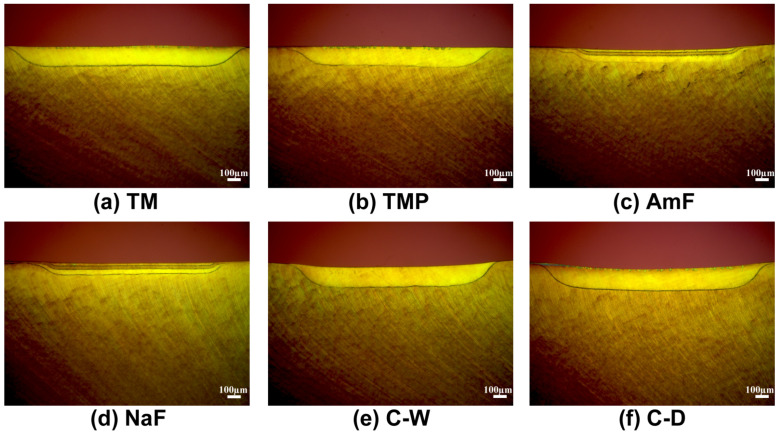
Polarized light images of dentin lesions showing different lesion depths caused by the protective influence of treatment with different toothpaste slurries (**a**–**d**) during artificial demineralization compared to controls (**e**,**f**).

The polarized light images (Figure 5 and Figure 6) represent characteristic signs of enamel and dentin lesions that were caused by artificial demineralization and the different treatment modalities of the test and control groups.

The morphological comparison of enamel lesions showed, that in groups TM (89%) and TMP (78%) (Figure 5a,b) a smaller number of severe lesions were observed in comparison to the negative controls C-W (100%) and C-D (95%) (Figure 5e,f). However, comparing test groups with the positive controls, only signs of demineralization (NaF: 100%/AmF: 76%) (Figure 5c,d) and moderate lesions (AmF: 24%) were detectable.

For group AmF, areas of increased delineation of enamel prisms and small areas with positive birefringence below a thick surface layer of 32 ± 8 µm were observed (Figure 5c). Signs of demineralization below a thick surface layer of 40 ± 9 µm are typical for group NaF (Figure 5d).

## 4. Discussion

Over the last few decades different new approaches have been discussed which focused on the prevention of dental caries and erosion [1,10,29,30].

This study demonstrates the demineralization protective effect of the CPP-products TM and TMP in comparison to the negative controls. These results confirm the effectiveness of CPP-ACP products that was also observed by other authors [31,32,33]. For example, in a systematic review by Ekambaram et al., it has been discussed, that CPP-ACP containing products have a higher enamel remineralization effect compared to other calcium- and phosphate-based agents [8]. Other authors were able to show a significant increase of microhardness in early enamel lesions of human premolars after artificial demineralization and treatment with CPP-ACP [8,10,31,32,33].

The use of human enamel and dentine specimens was considered relevant for the present investigation. Bovine enamel, such as used in other studies possesses certain advantages but has different properties particularly regarding lesion formation after demineralization compared to human enamel [34].

The demineralization solution used in this study was adjusted to a pH-value of 4 for several reasons: First, the optimal pH value for milk proteins to bind to dental hard tissues ranges from pH 3.5 to 5.5 [26,35]. Lower pH levels lead to a reduced chemical affinity. Furthermore, it was shown in another study that CPP-ACP/-ACFP possesses a distinct remineralization effect at a pH level between 4.5 and 5.5 [35].

Polarized light microscopy was used in the present investigation to assess the degree of demineralization of the enamel and dentine specimens. This method is well established in our group.

To compare CPP-ACP/-ACFP products concerning demineralization protective qualities, two commercially available fluoride toothpastes were used as positive controls [35].

The present study revealed that CPP-ACP-products TM and TMP were less effective compared to the fluoride containing toothpastes under the given conditions. Furthermore, the morphological aspects of the enamel lesions clearly underline the higher efficiency of control groups AmF and NaF. In the polarized light images (respective Figure) the severity of the enamel lesions in the TM/TMP and the control groups C-W/C-D can clearly be recognized by the pronounced dark body of the lesions. This typical lesion zone is clearly less pronounced in groups AmF and NaF. It should be noted that in the group NaF, four specimens with no signs of demineralization at all (complete remineralization) were found.

Those findings correspond to the generally accepted evidence on the effectiveness of fluoride in caries prevention [36,37,38].

The limited effect of CPP has also been discussed in other studies. In a systematic review it has been shown that there are no significant benefits of using CPP-ACP products over fluoride toothpaste for the prevention of early dental caries [15]. Other authors demonstrated that CPP-ACP might lead to accumulation of mineral components in the pellicle. However, they were not as effective as fluoride products [39].

Particular attention was paid to the TMP group since it combines two active components, fluoride and ACC-ACP. Possible synergistic effects of both substances are described in various studies [10,27]. In the present study the slight difference between the lesion depths of the TM and TMP groups was not significant (respective Figure 4).

The heterogeneous findings of other groups that investigated CPP-ACP products might be explained by the complexity of carious and erosive demineralization and the variety of application forms of CPP-ACP [40,41,42,43,44]. Another reason for the limited effectiveness of TM and TMP might be a chemical diffusion barrier, triggered by CPP-ACP/-ACFP.

In future studies, it should be investigated whether casein-based peptide complexes interact or congregate with proteins and other substances of the saliva or the dental pellicle layer. Both are involved in the de- and re-mineralization process of enamel and dentin. Also application parameters such as frequency and duration might influence the effectiveness of CPP-ACP products. In our study, 19 (nine for dentin) applications of Tooth Mousse were performed over a period of 14 days (seven for dentin). In contrast, a study by Miyahira et al. showed that demineralization of human enamel was inhibited more effective when CPP-ACP toothpastes were applies more frequently [41].

It can be concluded that under the given experimental conditions, CPP-ACP products (groups TM and TMP) possessed a significantly higher demineralization protective effect when compared to the negative control groups C-W (distilled water) and C-D (only demineralization, no treatment). However, regarding prevention of enamel and dentin demineralization, CPP-ACP test groups TM and TMP were less effective than fluoride toothpastes (positive groups AmF and NaF).

In the light of the controversial results obtained with TM products the anti-demineralization capability of CPP-ACP and CPP-ACFP should be further investigated using long term in vivo studies.

## Data Availability

Not applicable.
